# Expert consensus on endoscopic papillectomy using a Delphi process

**DOI:** 10.1016/j.gie.2021.04.009

**Published:** 2021-04-19

**Authors:** Jeska A. Fritzsche, Paul Fockens, Marc Barthet, Marco J. Bruno, David L. Carr-Locke, Guido Costamagna, Gregory A. Coté, Pierre H. Deprez, Marc Giovannini, Gregory B. Haber, Robert H. Hawes, Jong Jin Hyun, Takao Itoi, Eisuke Iwasaki, Leena Kylänpaä, Horst Neuhaus, Jeong Youp Park, D. Nageshwar Reddy, Arata Sakai, Michael J. Bourke, Rogier P. Voermans

**Affiliations:** 1 Department of Gastroenterology and Hepatology, Amsterdam University Medical Center, Amsterdam Gastroenterology Endocrinology & Metabolism, Amsterdam, the Netherlands; 2 Department of Hepatogastroenterology, Assistance Publique des Hôpitaux de Marseille, Aix-Marseille Université, Hôpital Nord, Marseille, France; 3 Department of Gastroenterology and Hepatology, Erasmus MC University Medical Center, Rotterdam, the Netherlands; 4 Department of Gastroenterology and Hepatology, Weill Cornell Medicine, New York Presbyterian Hospital, New York, New York, USA; 5 Digestive Endoscopy Unit, Fondazione Policlinico Universitario A. Gemelli, IRCCS, Catholic University, Rome, Italy; 6 Division of Gastroenterology and Hepatology, Department of Medicine, Medical University of South Carolina, Charleston, South Carolina, USA; 7 Department of Hepato-Gastroenterology, Cliniques universitaires Saint-Luc, Université Catholique de Louvain, Brussels, Belgium; 8 Endoscopic Unit, Paoli-Calmettes Institute, Marseille Cedex, France; 9 Divison of Gastroenterology and Hepatology, NYU Langone Medical Center, New York University, New York, New York, USA; 10 Center for Interventional Endoscopy, AdventHealth, Orlando, Florida, USA; 11 Division of Gastroenterology, Department of Internal Medicine, Korea University Ansan Hospital, Ansan, Korea; 12 Department of Gastroenterology and Hepatology, Tokyo Medical University, Tokyo, Japan; 13 Division of Gastroenterology and Hepatology, Department of Internal Medicine, Keio University School of Medicine, Tokyo, Japan; 14 Department of Gastrointestinal and General Surgery, Helsinki University Central Hospital, Helsinki, Finland; 15 Department of Gastroenterology, Evangelisches Krankenhaus Düsseldorf, Düsseldorf, Germany; 16 Department of Internal Medicine, Yonsei University College of Medicine, Seoul, Korea; 17 Department of Gastroenterology, Asian Institute of Gastroenterology Hospitals, Hyderabad, India; 18 Division of Gastroenterology, Department of Internal Medicine, Kobe University Graduate School of Medicine, Kobe, Japan; 19 Department of Gastroenterology and Hepatology, Westmead Hospital, and Westmead Clinical School, University of Sydney, Sydney, New South Wales, Australia

## Abstract

**Background and Aims:**

Consensus regarding an optimal algorithm for endoscopic treatment of papillary adenomas has not been established. We aimed to assess the existing degree of consensus among international experts and develop further concordance by means of a Delphi process.

**Methods:**

Fifty-two international experts in the field of endoscopic papillectomy were invited to participate. Data were collected between August and December 2019 using an online survey platform. Three rounds were conducted. Consensus was defined as ≥70% agreement.

**Results:**

Sixteen experts (31%) completed the full process, and consensus was achieved on 47 of the final 79 statements (59%). Diagnostic workup should include at least an upper endoscopy using a duodenoscope (100%) and biopsy sampling (94%). There should be selected use of additional abdominal imaging (75%−81%). Patients with (suspected) papillary malignancy or over 1 cm intraductal extension should be referred for surgical resection (76%). To prevent pancreatitis, rectal nonsteroidal anti-inflammatory drugs should be administered before resection (82%) and a pancreatic stent should be placed (100%). A biliary stent is indicated in case of ongoing bleeding from the papillary region (76%) or concerns for a (micro)perforation after resection (88%). Follow-up should be started 3 to 6 months after initial papillectomy and repeated every 6 to 12 months for at least 5 years (75%).

**Conclusions:**

This is the first step in developing an international consensus–based algorithm for endoscopic management of papillary adenomas. Surprisingly, in many areas consensus could not be achieved. These aspects should be the focus of future studies. (Gastrointest Endosc 2021;94:760–73.)

The first endoscopic papillectomy (EP) for papillary adenoma (n = 2) was described in 1983, and the first substantial cohort (n = 25) was published 10 years later.^1,2^ A number of predominantly retrospective case and cohort studies have been published subsequently.^3–7^ Despite the lack of randomized controlled trials and prospective series, EP for papillary adenoma is considered a relatively safe, minimally invasive treatment for lesions without significant intraductal extension or invasive disease.^5^ It has proven difficult to generate high-level scientific knowledge on the best treatment algorithm primarily because of low incidence and therefore difficult to conduct large prospective or randomized controlled studies. Subsequently, a consensus for EP practices has not been established. In 2015 the Standards of Practice Committee of the American Society for Gastrointestinal Endoscopy attempted to develop an evidence-based guideline on the role of endoscopy in papillary and duodenal adenomas.^8^ However, based on the current literature only limited recommendations regarding the optimal diagnostic workup, treatment, and follow-up protocol could be made. Unsurprisingly, at present there remains a wide variety in daily practice, mostly based on individual preferences.^9,10^

The aim of this study was to assess the level of consensus among international experts and to obtain further consensus by using a Delphi process. The Delphi method was developed by the Rand Corporation in the 1950s and was originally used in forecasting.^11^ Since then, the Delphi method is considered to be a reliable instrument to develop clinical consensus guidelines. This iterative structured process is widely used to achieve expert consensus for subjects where no definitive evidence is available and where expert opinion is important.^12–14^ The process consists of a series of questionnaires (usually 3), and after each round the responses are summarized and anonymously redistributed for discussion in the next round(s).^15^ Accordingly, the ultimate goal is to conduct a proposal for a more standardized treatment protocol on the performance of EP.

## METHODS

### Systematic literature search

A systematic search of literature was performed in PubMed and EMBASE databases on December 18, 2020. The search strategy can be found in Supplementary Figure 1 (available online at www.giejournal.org).

### Expert panel selection

Researchers were identified through authorship of relevant articles. Senior authors of original articles published in the last 15 years with a cohort of at least 20 patients were selected. Thus, 38 authors were invited. Furthermore, 14 longstanding and internationally recognized experts in the field of EP who did not appear in the search were selected to include broad clinical experience that might not be found in published literature. After the first round, only respondents who performed at least 30 EPs in their career were asked to join the consecutive rounds.

### Conduct of surveys

Three rounds were conducted. The first survey was based on systematic literature and personal experience in daily practice of the senior authors (M.J. Bourke and R. P. Voermans). The survey consisted of 54 multiple-choice questions divided over 6 different sections: background of the respondent, diagnostic workup, lesion assessment and staging, technical aspects, adverse events and their management, and follow-up. After each question there was an option to add other answer options or to share general comments on the question. The questions of which the answers reached consensus were transformed into statements and presented again to respondents in the next round. Questions without consensus were extracted, modified based on the comments of the respondents, and proposed again in the consecutive round(s). After each round responses were summarized and anonymously redistributed for discussion in the next round(s) to provide respondents the opportunity to review and possibly change their answers based on group consensus.

### Consensus development process

Data were collected between August and December 2019 using an online survey platform. The invited experts were given at least 2 weeks to complete the survey(s), with reminder e-mails sent twice during each completion period. Participants’ names and contact details were recorded to acknowledge their participation in the eventual article and to be able to ask them to participate in possible follow-up studies. However, participants were unaware of the names of other participants, and results were anonymized.

### Statistical analysis and grading of statements

Respondents were able to show their level of agreement with the proposed statements by scoring on a 5-point Likert-scale (1 = completely disagree, 2 = disagree, 3 = neutral, 4 = agree, 5 = completely agree) and could comment on their reason to agree or disagree.^16^ Consensus was defined as at least 70% agreement between the respondents (either (dis)agree or completely (dis)agree). Questions in which participants were asked to rank the answer possibilities were analyzed using median and interquartile range (IQR) in which an IQR ≤1 was considered consensus.^17,18^ Strength of the consensus statements was based on the level of evidence of the supporting literature according to the definitions of the Oxford Centre for Evidence-Based Medicine.^19^

## RESULTS

### Systematic review

After removing duplicates, 827 records were identified. Based on title and abstract screening, 627 records were excluded. Only an abstract was available for 92 records, resulting in 108 full-text articles that were screened for eligibility. The inclusion and exclusion process is summarized in Supplementary Figure 1. The relevant results are described and discussed together with the results of the Delphi process below.

### Participants

**Figure 1.**
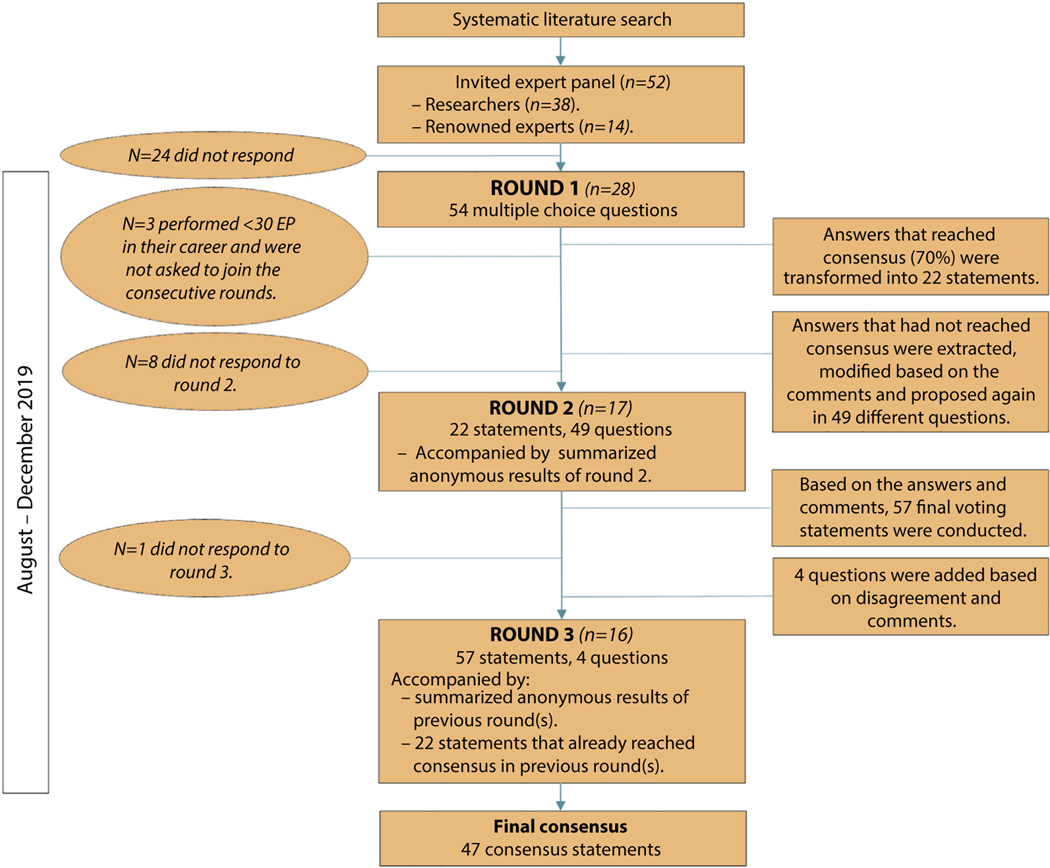
Flowchart study process. *EP*; Endoscopic papillectomy.

Fifty-two experts were invited. Twenty-eight (53.8% response rate) completed the first round, 17 (32.7% response rate) completed the second round, and 16 joined round 3 (30.7% response rate). Three participants of round 1 were not asked to join consecutive rounds because they had performed fewer than 30 EPs in their career. The 16 final participants included gastroenterologists from 10 different countries and 3 continents (Asia, North America, and Europe). Most participants (15 [94%]) primarily worked in a university hospital setting. All participants performed at least 30 EPs, and 11 (69%) had at least 20 years of experience. A summary of the study process is depicted in Figure 1.

### Recommendation statements

**Figure 2.**
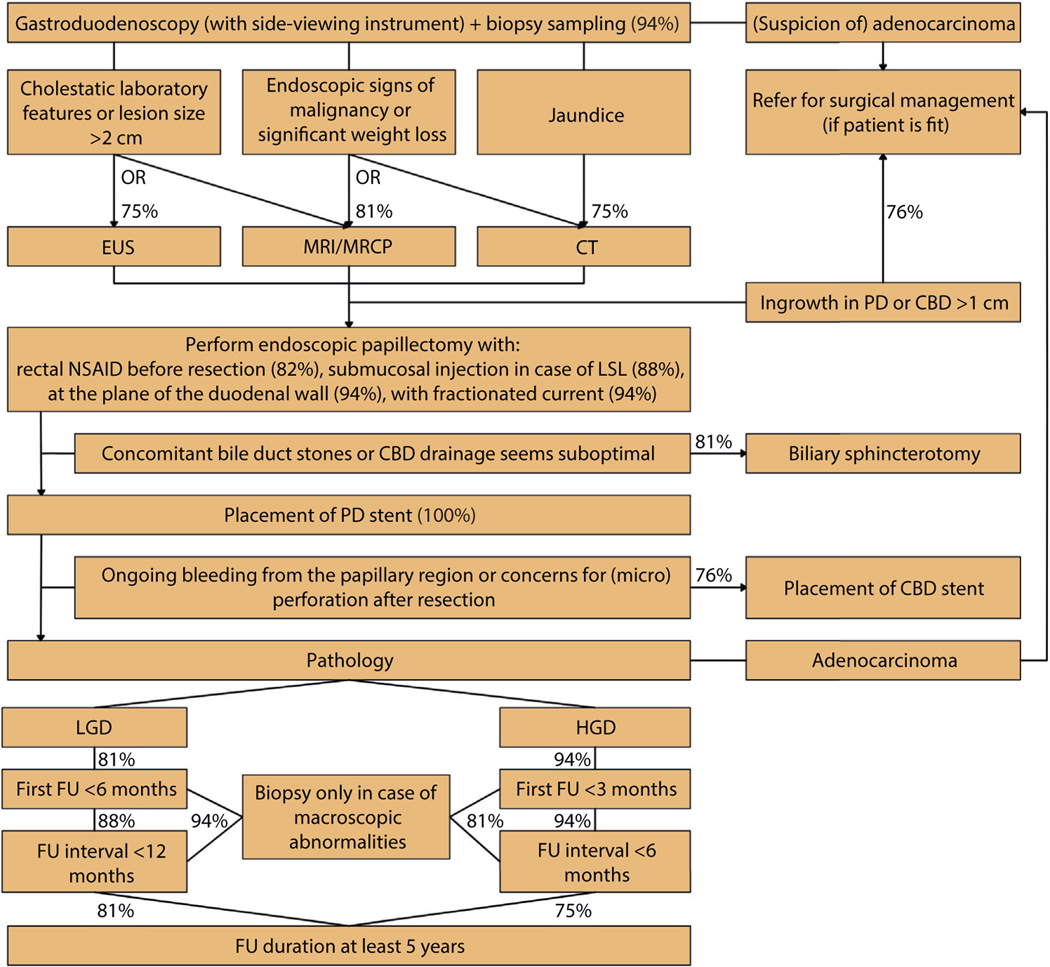
Consensus-based flowchart. Percentages indicate degree of agreement. *CBD*, Common bile duct; *CT*, computed tomography; *EUS*, endoscopic ultrasound; *FU*, follow-up; *HGD*, high-grade dysplasia; *LGD*, low-grade dysplasia; *LSL*, laterally spreading lesion; *MRCP*, magnetic resonance cholangiopancreatography; *MRI*, magnetic resonance imaging, *NSAID*, nonsteroidal anti-inflammatory drug; *PD*, pancreatic duct.

In the last round, a final 79 statements were proposed to the participants; the most important consensus statements are summarized in Table 1. Figure 2 depicts a consensus-based flowchart summarizing these statements. All statements that reached consensus are shown in Table 2. A selection of statements that did not reach consensus in the final round is shown in Table 3. The results of all 3 rounds are provided in Supplementary Tables 1–3 (available online at www.giejournal.org).

#### Diagnostic workup.

**Figure 3.**
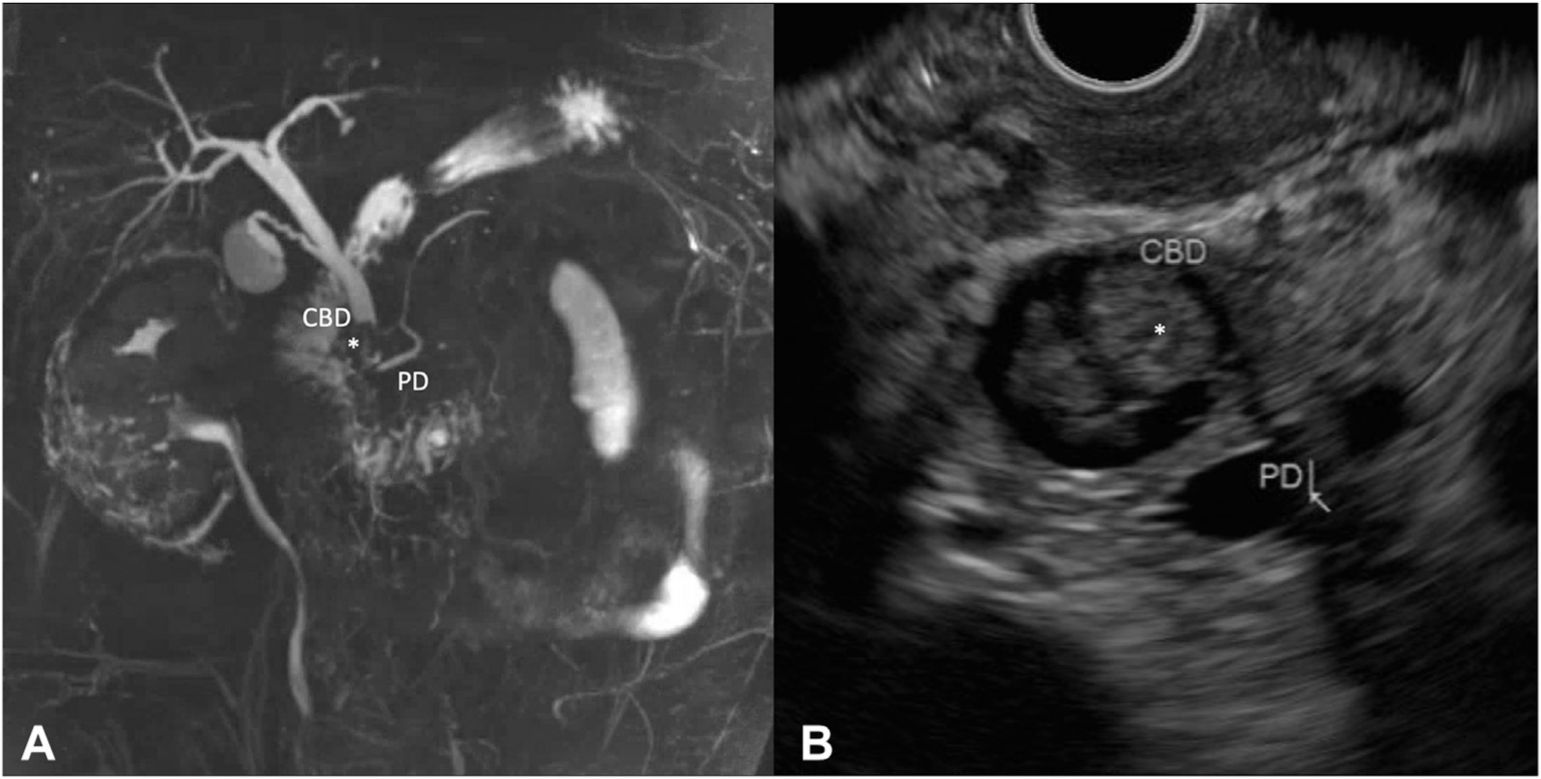
**A**, MRCP showing ingrowth (*) in the distal common bile duct (CBD) of approximately 15 mm. **B**, EUS showing ingrowth (*) in the distal CBD of approximately 12.5 mm and dilatation of both CBD (14.4 mm) and pancreatic duct (PD) (5.5 mm).

The standard diagnostic workup of a patient with a papillary lesion should include a gastroduodenoscopy with a side-viewing instrument (100%) and biopsy sampling (94%) before resection. Additional abdominal imaging should only be performed for specific indications (75%−81%). Magnetic resonance imaging (MRI)/magnetic resonance cholangiopancreatography (MRCP) or endoscopic ultrasound (EUS) can be used to rule out intraductal extension and should at least be performed in case of cholestatic laboratory features (81%) and/or lesion size >2 cm (75%) (Table 1). Figure 3 shows intraductal extension on MRI/MRCP and EUS. No consensus was reached that intraductal extension should be ruled out standardly in every patient before resection (53%) (Supplementary Table 2). MRI/MRCP or computed tomography (CT) should be performed in case of significant weight loss and/or endoscopic signs of malignancy. In case of jaundice a CT should be performed as well (Table 1).

The only existing international guideline in EP from 2015 states that endoscopic retrograde cholangiopancreatography (ERCP) should be performed in every patient at the time of resection to assess for evidence of intraductal extension. ^8^ However, this was shown not to be common practice, with only 25% of respondents considering endoscopic cholangiography as part of the standard diagnostic workup (Supplementary Table 1). Our study does agree with the previous guideline statement that biopsy sampling should be performed in every patient before resection (94%). Nevertheless, the accuracy of biopsy sampling in papillary lesions is questionable; according to pathology studies preresection biopsy sampling accurately diagnosed 70% of papillary malignancies^20–22^ compared with at least 80% in the colon.^23^ Biopsy sampling is also important when the diagnosis of adenoma is considered in a larger than normal papilla.

#### Lesion assessment.

Because the accuracy of biopsy sampling in papillary lesions is questionable, careful endoscopic assessment is considered most important. However, final pathology may differ from the initial endoscopic diagnosis. Limited data of patients suspected to have a benign adenoma based on endoscopic appearance indicate that final pathology shows another diagnosis (such as normal mucosa, gastric heterotopia, adenomyomatosis, hamartoma, neuroendocrine tumor, or adenocarcinoma) in 10% to 20%.^6,24,25^ Furthermore, a recent series of patients who underwent EP showed that in only half of the lesions in which the resection specimen showed adenocarcinoma, malignancy was already suspected based on the endoscopic appearance, further questioning the accuracy of endoscopic assessment.^26^

Advanced imaging techniques such as narrow-band imaging and chromoendoscopy have proven to be a valuable addition to white-light imaging in the diagnosis of colorectal lesions.^27^ However, the possible benefit of narrow-band imaging in the assessment of papillary lesions is only described in a small case series.^28^ Accordingly, there is consensus that these techniques are not helpful in distinguishing between benign and malignant papillary lesions at this moment (71%) (Table 2).

Despite the current lack of a predefined classification system to determine whether a papillary lesion is most likely benign or malignant (89%) (Table 2), consensus exists that features such as ulceration (median, 4; IQR, 1) and immobility (median, 4; IQR, 1) should be considered features of a potential malignant lesion, regardless of biopsy sample results (Supplementary Table 2). When ulceration is present, this lesion can even be defined as most likely malignant based on this sole feature, regardless of biopsy sample results (94%) (Table 1). In case the biopsy sample shows high-grade dysplasia, firm consistency is considered an important characteristic as well (median, 4; IQR, 1). Features such as smooth or irregular surface, tumor size >4 cm, spontaneous bleeding, and excessive friability are, as sole criteria, of less importance when assessing a papillary lesion (Supplementary Table 2).

#### Patient selection.

Although certain features would define a lesion as most likely malignant, none of the mentioned characteristics should be considered as an independent reason to refer for surgical management (Supplementary Table 2).

Considering the risk of incomplete endoscopic resection, consensus exists that patients should be referred for surgical management when ingrowth in the pancreatic duct (PD) or common bile duct (CBD) of >1 cm is present (76%) (Table 1). Jaundice (86%), ingrowth in the PD (79%) or CBD ≤1 cm (86%), and classification as an umbilicated lesion (a sign of central retraction) (82%) are not considered independent reasons to refer the patient for surgical management (Table 2). Nonetheless, no consensus exists on which additional techniques should be used to endoscopically resect ingrowth in the CBD or PD ≤1 cm (Supplementary Table 2–3). Furthermore, no agreement exists that intraductal extension should be ruled out routinely before resection (53%) (Supplementary Table 2).

Moreover, in case a patient is unfit for surgery, endoscopic resection can, if technically feasible, still be considered in case of adenocarcinoma (75%) or ingrowth in the CBD >1 cm (81%) (Table 2). Additionally, intraductal radiofrequency ablation (RFA) has been successfully described in small studies.^29–31^ Accordingly, experts agree that EP with the additional use of RFA can be considered in a patient who is not a surgical candidate when ingrowth in the CBD >1 cm is present (75%) (Table 1). However, no consensus exists whether to consider endoscopic resection in combination with RFA when surgery is still an option (44%) (Supplementary Table 3).

#### Technical aspects.

Resection should be performed at the plane of the duodenal wall (94%) with fractionated current (short, regular pulses of cutting current integrated in background of coagulation current), regardless of the size of the lesion (94%) (Table 2). A systematic review comparing fractionated and cutting current showed no difference in adverse events. This result was confirmed by a small randomized controlled pilot study that showed no difference in terms of safety and efficacy. Although the use of fractionated current might prevent immediate bleeding in larger adenomas, it may cause crush artefacts.^32,33^

Pancreatic and biliary sphincterotomy should, if indicated, only be performed after resection (100%) (Table 2). Indications for biliary sphincterotomy are concomitant bile duct stones or suboptimal drainage (81%) (Table 1). No consensus exists on indications for pancreatic sphincterotomy (38%−44%) (Table 3).

Submucosal injection should in general only be performed in case of a laterally spreading lesion (88%) (Table 1). This statement is confirmed by a small randomized controlled trial that could not show advantages in the use of submucosal injection and concluded that resection without lifting would be simpler and therefore primarily the recommended technique.^34,35^

The effect of preventive PD stent placement on postpapillectomy pancreatitis (PPP) has been claimed by 1 small randomized controlled trial. However, this difference was only significant in the per-protocol analysis but not in the intention-to-treat analysis.^36^ Nevertheless, more recently 2 systematic reviews and meta-analyses of available literature supported the preventive effect of PD stent placement as well.^37,38^ Accordingly, experts agree on the routine use of a PD stent to prevent PPP (100%) (Table 1). Different methods of PD stent placement have been studied; for example cannulating the PD before resection and performing resection with the guidewire in situ has been suggested. Because this method could potentially hamper complete en-bloc resection, consensus was achieved that the PD should be cannulated after resection (100%) (Table 2).^39–42^ However, it remains unclear if injecting the PD before resection would be helpful in finding the PD after resection (44%) (Table 3).^43,44^ Moreover, there was no consensus regarding the use of a PD stent with or without an internal flap (46% vs 54%) (Supplementary Tables 1–2).

No consensus exists that pancreas divisum should be routinely excluded before resection (65%) (Supplementary Table 2). In the final round only 63% agreed that either EUS or MRI/MRCP should be performed in every patient before resection (Table 3).

A CBD stent should only be placed on indication (82%) (Table 1). No consensus was reached on the standard placement of a stent inside the CBD (18%) (Supplementary Table 1) to, for example, prevent postintervention cholangitis, which has been described in 0% to 7% of cases.^3,5,26^ Possible indications to place a CBD stent are concerns for a (micro)perforation (88%) or ongoing bleeding from the papillary region during the procedure (76%). In case of concerns for a (micro) perforation, a fully covered self-expanding metal stent (FCSEMS) is preferred over a plastic stent (88%) (Table 1). FCSEMSs could also be useful in case of bleeding from the biliary region by tamponading the bleeding vessel.^45^ Remarkably, no consensus was reached that a FCSEMS was preferred in case of bleeding as well, probably because the statement did not incorporate bleeding from the papillary region (63%) (Table 3). In case of residual tissue, stent placement could also facilitate the direct inspection of the distal CBD in the first follow-up procedure to exclude and treat possible intraductal extension.^4^ Nonetheless, no consensus was achieved on the treatment of residual adenomatous tissue and the use of FCSEMSs in this manner (31%) (Table 3).

Snare tip soft coagulation of the resection margins after endoscopic mucosal resection (EMR) of adenomas in the colon results in a 4-fold reduction in recurrence at first follow-up.^46^ Recent (preliminary) data show similar results after EMR of duodenal adenomas, suggesting that snare tip soft coagulation could also prevent recurrence after EP.^47^ However, considering the potential risk of perforation and/or pancreatitis this technique is, reasonably, not part of current practice at the moment (56%), and further prospective study is required to evaluate its potential utility (Table 3).

Prophylactic clip closure of the mucosal defect to prevent bleeding has been successfully described after endoscopic resection of large colon polyps.^48^ Also, a recent small prospective study examined the preventive closure of the frenulum after EP, which led to a decrease in delayed bleeding without a shown increase in pancreatitis or perforation rates and without lengthening procedure time.^49^ However, data are limited, and there is no agreement whether standard clip closure of the mucosal defect after resection should be performed (38%) (Table 3).

**Figure 4.**
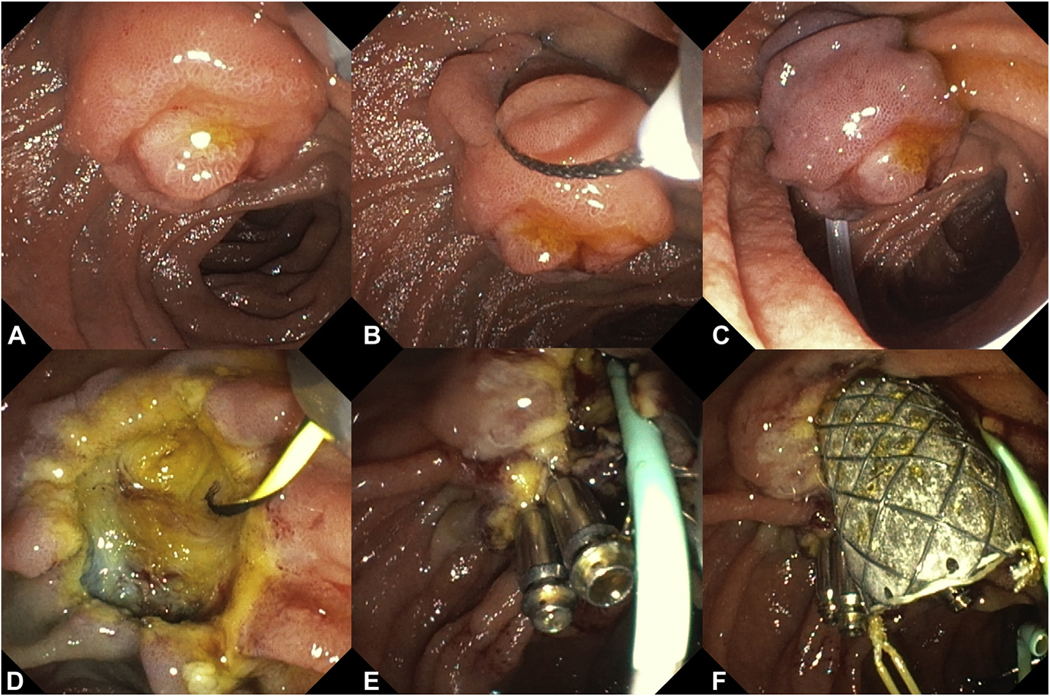
**A-C**, Conventional en-bloc papillectomy at the level of the duodenal wall for 15 mm papillary adenoma. **D-F**, Exposure of biliary and pancreatic orifices with a 5F pancreatic stent and fully covered metal biliary stent. Clip closure of the frenulum for the prophylaxis of post-papillectomy bleeding.

**Figure 5.**
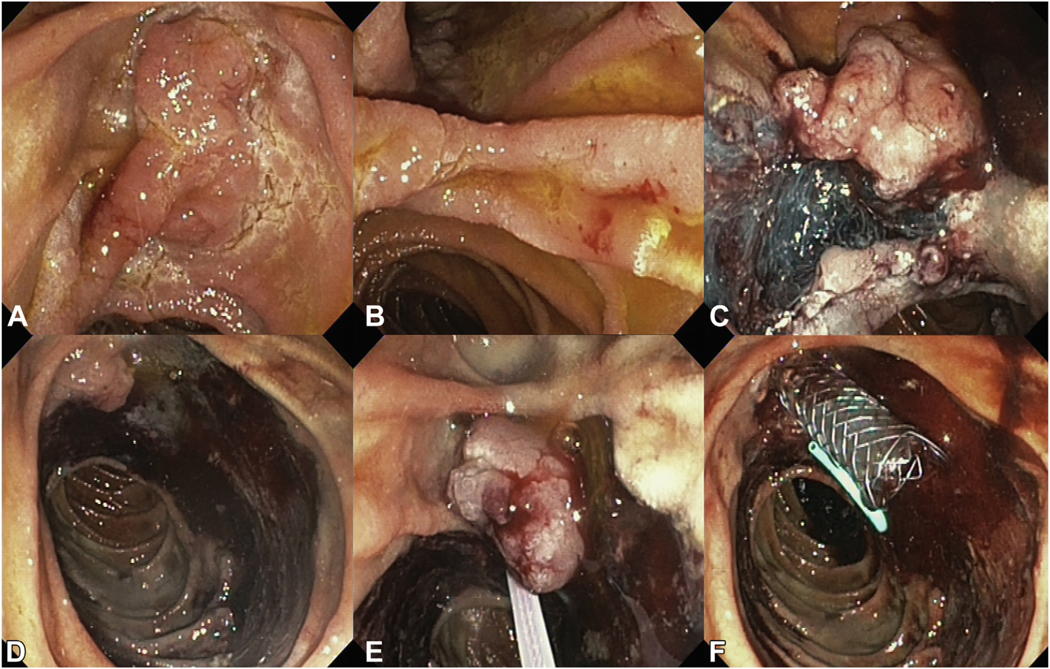
**A** and **B,** Extensive laterally spreading papillary adenoma involving greater than two-thirds of the duodenal circumference. **C** and **D,** Piecemeal EMR of the laterally spreading components resulting in 90% circumferential mucosal defect. **E** and **F,** En-bloc papillectomy followed by a 5F pancreatic stent and fully covered metal biliary stent.

The use of antispasmodic drugs such as glucagon and scopolaminebutyl can be helpful in reducing the risk of losing the specimen distally into the small bowel; however, no consensus exists whether to administer these drugs routinely before resection (56%) (Table 3). Probably because, in case of an adequate complete prone position of the patient, the risk of losing a specimen is considered low because it will generally migrate proximally with gravity toward the duodenal bulb. Figures 4 and 5 show the removal of a conventional and an extensive laterally spreading papillary adenoma, respectively, including placement of a PD and CBD stent, en-bloc removal of the adenoma at the plane of the duodenal wall, and clipping of the frenulum.

#### Adverse events and management.

In addition to the placement of a PD stent after resection, rectal nonsteroidal anti-inflammatory drugs should be given before resection to further aid in the prevention of PPP (82%) (Table 1). This consensus statement is supported by studies regarding pancreatitis prevention in conventional ERCP practice that showed a significant reduction in incidence when using nonsteroidal anti-inflammatory drug suppositories.^50–52^ In addition, studies suggest that preventive vigorous pre- and perprocedural hydration reduces the pancreatitis rate even further.^53–55^ This outcome still needs to be confirmed by an adequately powered randomized controlled trial of which the results have not yet been published.^56^ Accordingly, no consensus could be reached that vigorous hydration should be considered in, for example, cases without any major cardiac comorbidity (63%) (Table 3).

Consensus could not be achieved on whether patients should be treated with a proton pump inhibitor (PPI) to decrease the risk of delayed bleeding after performing an EP (69%) (Table 3). Although the effect of acid suppression by a PPI in the treatment of upper GI tract bleeding caused by (artificial) ulcers is evident, the benefit of PPI use in the prevention of delayed bleeding after EP or, for example, sphincterotomy is not established and questionable given the relatively high pH in the duodenum.^57,58^

Considering bleeding, different methods were proposed to stop the bleeding such as the use of FCSEMSs, Hemospray (Cook Medical, Bloomington, Ind, USA), epinephrine injection, clips, or coagulation. No consensus could be achieved on the best treatment method in case of bleeding during the procedure (Supplementary Tables 2–3). Furthermore, no consensus seems to exist as to whether to perform reintervention (38%) or initially treat a patient conservatively (63%) in case of delayed bleeding when the patient is hemodynamically stable after resuscitation (Table 3).

#### Follow-up.

Biopsy sampling during follow-up only needs to be performed when macroscopic abnormalities are present (81%). The first follow-up should be performed within 3 months (94%) with an interval to the second follow-up of 6 months or less (94%) in case of high-grade dysplasia compared with, respectively, 6 (81%) and 12 months (88%) in case of low-grade dysplasia. In both cases follow-up should be continued for at least 5 years (75%−81%) (Table 2); however, 31–38% of experts would perform lifelong follow-up, as long as the patient is fit (Supplementary Table 3). Recent data on long-term follow-up after EP show that recurrence has been found even 5 years after the index procedure, confirming the need for consideration of longer follow-up.^7,26^

Finally, there was consensus that patients should be admitted for observation after the procedure (82%) (Supplementary Table 2). However, participants could not agree on the length of necessary observation, 69% would observe at least 24 hours, and 44% at least 48 hours (Table 3).

## DISCUSSION

EP is established as the preferred method to manage benign papillary adenomas.^5,59^ EP is minimally invasive, and modeling using well-validated scoring systems has shown it is safer and less expensive than surgical management. ^60^ Moreover, in case of unsuspected cancer in the papillectomy specimen, it does not preclude or compromise subsequent surgery in a surgically fit patient. Although generally considered safe, EP is not without risks, with serious adverse events occurring in 15–35% of patients and recurrence in up to 20% during surveillance.^3–7,26^ It is therefore incumbent on those managing these patients to further refine and optimize the EP procedure to mitigate against these adverse events. However, high-level scientific knowledge to guide this process is largely absent because papillary adenomas are uncommon, and thus large prospective multicenter or randomized controlled studies have not been executed. Therefore, unsurprisingly, an evidence-based consensus for EP has not been established. We sought to address this deficiency by using a Delphi process among international experts.

Sixteen international experts joined the final round, and consensus was reached on 47 of the final 79 proposed statements. It was necessary to propose a large number of statements to fully evaluate this complex multifaceted procedure. Based on the consensus achieved, insight was given in the main characteristics that should be taken into account when classifying a papillary lesion and reasons to refer a patient for surgical management. Furthermore, a consensus-based algorithm regarding diagnostic workup, technical aspects, and follow-up is proposed and is depicted in Figure 2.

As stated, biopsy sampling is only accurate in 70% of the papillary malignancies, and therefore endoscopic assessment can be considered most important.^20–22^ To improve this classification, it would be helpful to have a predefined scoring system to better assess these lesions. This does not currently exist, and although we were able to identify important characteristics deemed to be associated with malignancy, further study is needed to propose a useful scoring system to improve the assessment of papillary lesions. Also, advanced imaging techniques such as narrow-band imaging or chromoendoscopy could be further assessed to determine their possible additional value to the classification of papillary lesions.

Besides the need for studies regarding optimal diagnosis, a lack of consensus on different technical aspects of the procedure warrants future prospective studies. For example, the benefit of FCSEMS placement after resection in case of bleeding from the papillary region or when residual tissue is present, RFA in case of ingrowth in the CBD, and the use of snare tip soft coagulation coagulation of the margins of the laterally spreading segment need to be further evaluated to determine their utility in daily practice. Furthermore, preventive measures such as vigorous hydration to prevent PPP and PPI to prevent delayed bleeding after resection are not part of the proposed consensus algorithm because, due to the paucity of data, no consensus could be reached on the benefit of these methods, showing the need for future well-targeted studies.

This study is not free of limitations. According to the low patient numbers in the literature and the absence of systematic prospective studies, high-level scientific enquiry to inform evidence-based consensus is lacking. Consequently and unsurprisingly, because of the lack of consensus on certain critical steps in the process, it is impossible to propose a complete algorithm for EP. In addition, note should be taken that the proposed protocol in this study mainly focuses on patients with sporadic papillary adenoma and is not directly translatable to patients with a genetic predisposition as in familial adenomatous polyposis syndrome. Although the resection technique and the prevention and treatment of adverse events can be considered the same, the diagnostic and follow-up protocol contain important differences. Final limitations are inherent to the study design; the expert panel, although reliant on published literature, was partly composed on personal expert knowledge, which makes the study eligible for personal bias. Because an adequate sample of possible participants was not available, test–retest reliability could not be performed, and, most importantly, when consensus was reached, this does not certainly mean that the correct answer has been found.^15^

However, given the lack of large prospective, randomized studies, this Delphi consensus provides the best available evidence regarding the management of these relatively uncommon lesions. The invited expert panel can be considered an adequate reflection of the experience in EP because researchers and renowned experts (therefore, wide clinical and scientific experience) were included. Furthermore, the size of the panel can be considered reliable for content validation because 5 to 10 experts are considered sufficient.^61^ By using a Delphi process, consensus could be achieved in a group of geographically spread experts, obviating the need for direct confrontation, leaving room for individual thought.^11^ The use of anonymous group feedback gave participants the opportunity to change their opinion based on group consensus. Moreover, consensus statements were supported by available literature after systematic review of the literature. Accordingly, this study provides a unique agreement and the best available, evidence-based guideline for the endoscopic management of papillary adenomas.

In conclusion, this Delphi study provides the current highest level of evidence regarding the different aspects of the performance of EP. Although there are surprisingly many areas in which no consensus exists and scientific data are lacking, this study led to the first consensus-based management algorithm for papillary adenomas. Therefore, this study can be considered a vital step around which future studies can be designed to ultimately generate a more robust evidence-based consensus guideline for EP. Furthermore, important insights were observed in areas in which a high variety in daily practice still exists, and, accordingly, future studies could be targeted.

## Supplementary Material

1

## Figures and Tables

**TABLE 1.** Important consensus statements

Statement	Agreement	Grading*
Diagnostic workup		

1. Gastroduodenoscopy with side-viewing instrument should always be performed before resection.	100%	D

2. Biopsy sampling should always be performed before resection.	94%	D

3. Either MRI/MRCP or EUS should be performed in case of a lesion larger than 2 cm and/or in case of cholestatic laboratory features.	75%	D

4. Either MRI/MRCP or CT should be performed in case of significant weight loss and/or in case of endoscopic signs of malignancy.	81%	D

5. CT should be performed in case of jaundice.	75%	D

Lesion assessment and staging		

6. When a lesion shows ulceration, this lesion should be defined as most likely malignant.	94%	D

7. Patient should be referred for surgical management in the following cases, considering patient is suitable for surgery:		

a. Ingrowth in the PD >1 cm.	76%	D

b. Ingrowth in the CBD >1 cm.	81%	D

8. If there is ingrowth in the CBD >1 cm, endoscopic papillectomy with radiofrequency ablation can be considered in a patient who is not a surgical candidate because of age and/or comorbidity, considering the lesion seems favorable for endoscopic resection.	75%	C

Technical aspects		

9. Submucosal injection should only be performed in case of a laterally spreading lesion.	88%	C

10. PD stent should be routinely placed to prevent postintervention pancreatitis.	100%	B

11. CBD stent should only be placed on indication, namely	82%	D

a. If there are concerns for a perforation in the papillary region after resection, a fully covered self-expanding metal stent should be placed in the CBD.	88%	D

b. In case of bleeding from the papillary region during the procedure.	76%	D

12. Biliary sphincterotomy should be performed in case of concomitant bile duct stones and in case drainage is deemed suboptimal.	81%	D

Adverse events and management		

13. Rectal nonsteroidal anti-inflammatory drugs should be given before resection.	82%	B

Follow-up		

14. In case initial pathology shows low-grade dysplasia, first follow-up (after removal of possible placed stents) should be performed within 6 months.	81%	D

15. In case initial pathology shows high-grade dysplasia, first follow-up (after removal of possible placed stents) should be performed within 3 months.	94%	D

16. Follow-up should be performed for at least 5 years.	75%	D

*CBD*, Common bile duct; *CT*, computed tomography; *EUS*, endoscopic ultrasound; *MRCP*, magnetic resonance cholangiopancreatography; *MRI*, magnetic resonance imaging; *PD*, pancreatic duct.

* Grading: A, level 1a-1b evidence; B, level 2a-3b evidence; C, level 4 evidence; D, level 5 evidence.

**TABLE 2.** Final consensus statements

Statement	Agreement	Grading*
Diagnostic workup		

1. Gastroduodenoscopy with side-viewing instrument should always be performed before resection.	100%	D

2. Advanced imaging techniques (such as narrow-band imaging or chromoendoscopy) are *not* helpful to distinguish between benign and malignant lesions.	71%	D

3. Biopsy sampling should always be performed before resection.	94%	D

4. Either MRI/MRCP or EUS should be performed in case of cholestatic laboratory features with or without jaundice.	81%	D

5. Either CT, MRI/MRCP, or EUS should be performed in case of cholestatic laboratory features with or without jaundice.	75%	D

6. CT should be performed in case of jaundice.	75%	D

7. Either MRI/MRCP or EUS should be performed in case of a lesion larger than 2 cm.	75%	D

8. Either MRI/MRCP or CT should be performed in case of significant weight loss.	81%	D

9. Either MRI/MRCP or CT should be performed in case of endoscopic signs of malignancy.	81%	D

Lesion assessment and staging		

10. No predefined classification system to determine if a papillary adenoma is most likely benign or malignant exists.	89%	D

11. When a lesion shows ulceration, this lesion should be defined as most likely malignant.	94%	D

12. The following characteristics are *not* a sole reason to define the lesion as most likely malignant:		

a. Smooth surface	96%	D

b. Spontaneous bleeding	86%	D

c. Lesion size >4 cm	86%	D

13. Patient should be referred for surgical management in case of ingrowth in the PD >1 cm, considering patient is suitable for surgery.	76%	D

14. Patient should be referred for surgical management in case of ingrowth in the CBD >1 cm, considering patient is suitable for surgery.	81%	D

15. The following situations are *not* a sole reason to refer for surgical management:		

a. Jaundice	86%	D

b. Ingrowth in the PD ≤1 cm	79%	D

c. Ingrowth in the CBD ≤1 cm	86%	D

d. An umbilicated lesion	82%	D

16. If biopsy sample shows LGD and ulceration is present, the lesion could still be resected endoscopically; there is no need to refer the patient for surgical management based on this sole characteristic, considering the lesion seems favorable for endoscopic resection.	88%	D

17. If there is ingrowth in the CBD >1 cm, endoscopic resection can still be considered if the patient is not a surgical candidate because of age and/or comorbidity, considering the lesion seems favorable for endoscopic resection.	81%	D

18. If there is ingrowth in the CBD >1 cm, EP with radiofrequency ablation can be considered in a patient that is not a surgical candidate because of age and/or comorbidity, considering the lesion seems favorable for endoscopic resection.	75%	C

19. If biopsy sample shows adenocarcinoma in situ or well-differentiated adenocarcinoma, endoscopic resection can still be considered if the patient is not a surgical candidate because of age and/or comorbidity, considering the lesion seems favorable for endoscopic resection.	75%	D

Technical aspects		

20. Submucosal injection should only be performed in case of a laterally spreading lesion.	88%	C

21. Resection of the lesion should be performed at the plane of the duodenal wall.	94%	D

22. EP should be performed with fractionated current.	94%	D

23. If pancreatic sphincterotomy is indicated, then it should be performed after resection.	88%	D

24. Biliary sphincterotomy should be performed in case of concomitant bile duct stones and in case drainage is deemed suboptimal.	81%	D

25. If biliary sphincterotomy is indicated, then it should be performed after resection.	100%	D

26. PD stent should be routinely placed to prevent postintervention pancreatitis.	100%	B

27. PD should be cannulated after resection.	100%	D

28. CBD stent should only be placed on indication, namely	82%	D

a. If there are concerns for microperforations in the papillary region after resection.	88%	D

b. In case of bleeding from the papillary region during the procedure.	76%	D

29. In case there are concerns for microperforations in the papillary region a fully covered self- expanding metal stent should be placed in the CBD.	88%	D

Adverse events and management		

30. Rectal nonsteroidal anti-inflammatory drugs should be given before resection.	82%	B

Follow-up		

31. In case initial pathology shows LGD		

a. First follow-up (after removal of possible placed stents) should be performed within 6 months.	81%	D

b. At first follow-up, biopsy specimens should only be taken when macroscopic abnormalities are present.	94%	D

c. Follow-up interval should be 12 months or less.	88%	D

d. At further follow-up, biopsy specimens should only be taken when macroscopic abnormalities are present.	94%	D

e. Follow-up should be performed for at least 5 years.	81%	D

32. In case initial pathology shows HGD		

a. First follow-up (after removal of possible placed stents) should be performed within 3 months.	94%	D

b. At first follow-up, biopsy specimens should only be taken when macroscopic abnormalities are present.	81%	D

c. Follow-up interval should be 6 months or less.	94%	D

d. At further follow-up, biopsy specimens should only be taken when macroscopic abnormalities are present.	81%	D

e. Follow-up should be performed for at least 5 years.	75%	D

*CBD*, Common bile duct; *CT*, computed tomography; *EUS*, endoscopic ultrasound; *EP*, endoscopic papillectomy; *HGD*, high-grade dysplasia; *LGD*, low-grade dysplasia; *MRCP*, magnetic resonance cholangiopancreatography; *MRI*, magnetic resonance imaging; *PD*, pancreatic duct.

* Grading: A, level 1a-1b evidence; B, level 2a-3b evidence; C, level 4 evidence; D, level 5 evidence.

**TABLE 3.** Selection of final round statements that did not reach consensus

Statement	Agreement
Diagnostic workup	

1. Either MRI/MRCP or EUS should be performed in every patient before resection.	63%

2. An endoscopic cholangiogram either before or during EP should only be performed if other performed tests are found inconclusive and there is still doubt about the presence of intraductal extension.	44%

Technical aspects	

3. STSC of the margins should *not* be performed after EP.	56%

4. STSC can be performed for the margins of the laterally spreading component but not the papillary margins.	50%

5. Pancreatic sphincterotomy after resection should only be performed in case of	

a. Extension in the pancreatic duct.	38%

b. Extension in the pancreatic duct or if drainage is deemed suboptimal.	44%

6. It can be helpful to inject the PD before resection to make it easier to find the PD after resection in case of extension in the pancreatic duct.	44%

7. In case there is bleeding during the procedure, an FCSEMS instead of a plastic stent should be placed in the CBD.	63%

8. In case there are concerns for residual adenomatous tissue in the distal part of the CBD, an FCSEMS should be placed in the CBD.	31%

9. Standard clip closure of the mucosal defect after resection should *not* be performed.	38%

10. Glucagon or scopulaminebutyl should be provided routinely before resection to reduce the risk of losing the specimen in the GI tract.	56%

Adverse events and management	

11. Vigorous hydration should be considered in patients without any cardiac comorbidity to further decrease the risk of postintervention pancreatitis.	63%

12. Every patient should be treated with PPI after performing an EP.	69%

13. Patients treated with PPI after resection should be treated for at least 2 weeks.	69%

14. If a bleeding occurs after EP and patient is hemodynamically stable after resuscitation with <1.2 mmol/L drop in hemoglobin	

a. Reintervention should be performed within 12 hours.	38%

b. Conservative treatment (continue or start PPI) is initially indicated.	63%

Follow-up	

15. Every patient should be admitted for observation after EP for	

a. At least 24 hours.	69%

b. At least 48 hours.	44%

*CBD*, Common bile duct; *EP*, endoscopic papillectomy; *EUS*, endoscopic ultrasound; *FCSEMS*, fully covered self-expanding metal stent; *MRCP*, magnetic resonance cholangiopancreatography; *MRI*, magnetic resonance imaging; *PD*, pancreatic duct; *PPI*, proton pump inhibitor; *STSC*, snare tip soft coagulation.

## Abbreviations:

CBD: common bile duct
CT: computed tomography
EP: endoscopic papillectomy
EMR: endoscopic mucosal resection
ERCP: endoscopic retrograde cholangiopancreatograpy
EUS: endoscopic ultrasound
FCSEMS: fully covered self-expanding metal stent
IQR: interquartile range
MRCP: magnetic resonance cholangiopancreatography
MRI: magnetic resonance imaging
PD: pancreatic duct
PPP: postpapillectomy pancreatitis
PPI: proton pump inhibitor
RFA: radiofrequency ablation
