# Supplementary material for: Expert consensus on endoscopic papillectomy using a Delphi process
Source: Gastrointest Endosc. Author manuscript; Available in PMC 2022 Feb 25. (PMC8878358; doi:10.1016/j.gie.2021.04.009)
Supplement: 1 [file NIHMS1757345-supplement-1.pdf]

APPENDIX

EMBASE (OVID)

Database(s): Embase Classic+Embase 1947 to 2020 December 17

#	Searches	Results
1	(endoscopic ampullectom* or papillectom*).ti,ab,kw.	692
2	((endoscopic resect* or endoscopic excision*) and (papilla* or ampulla*)).ti,ab,kw.	573
3	1 or 2	1114
4	limit 3 to (conference abstract status and yr="1883 - 2014")	194
5	3 or 4	920
6	limit 5 to English language	**782**

PubMed

Search performed on 2020 December 18

#	Searches	Results
1	(endoscopic resect*[tiab] OR endoscopic excision*[tiab]) AND (papilla*[tiab] OR ampulla*[tiab])	262
2	(endoscopic ampullectom*[tiab] OR papillectom*[tiab])	326
3	1 OR 2	**525**

Flowchart in- and exclusion

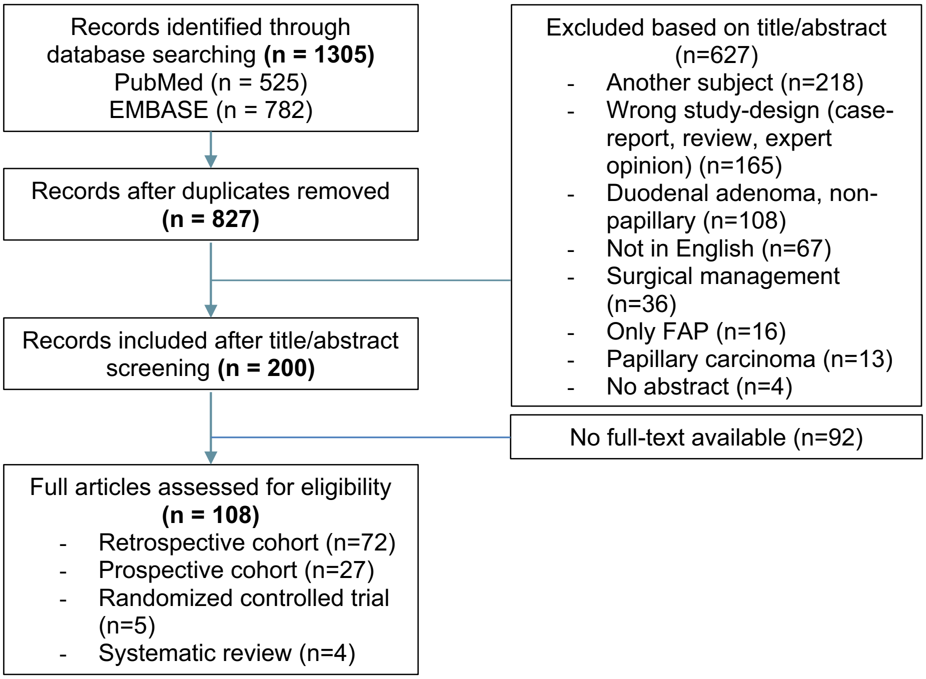

Supplementary Figure 1. Literature search.

SUPPLEMENTARY TABLE 1. Survey round 1

Question	Agreement
Expertise	
1. In which country do you work?	
a. United States of America	29%
b. Japan	14%
c. France	11%
d. Korea	11%
e. Germany	7%
f. Italy	7%
g. Netherlands	7%
h. Belgium	4%
i. Finland	4%
j. Switzerland	4%
k. United Kingdom	4%
2. In which type of practice do you primarily work?	
a. Academic practice	86%
b. Private practice	11%
c. Nonacademic practice	7%
d. Other	4%
3. How many years have you been in practice?	
a. <5 years	0%
b. 5-10 years	7%
c. 10-20 years	25%
d. >20 years	68%
4. Do you perform colonoscopies every week?	
a. Yes	82%
b. No	18%
5. How many colonoscopies do you perform every week? (n = 23)	
a. <5 per week	30%
b. 5-10 per week	35%
c. 10-20 per week	22%
d. >20 per week	13%
6. Do you perform basic polypectomy?	
a. Yes	93%
b. No	7%
7. Do you perform colonic EMR?	
a. Yes	82%
b. No	18%
8. How many ERCPs do you perform yearly?	
a. <100 per year	4%
b. 100-200 per year	21%
c. 200-400 per year	36%
d. >400 per year	39%

(*continued on the next page*)

SUPPLEMENTARY TABLE 1. Continued

Question	Agreement
9. How many endoscopic papillectomies are performed yearly in your center?	
a. <5 per year	4%
b. 5-10 per year	18%
c. 10-20 per year	36%
d. >20 per year	43%
10. How many endoscopic papillectomies did you perform in your career?	
a. <5 in total	0%
b. 5-10 in total	4%
c. 10-30 in total	7%
d. >30 in total	89%
11. Do you perform EUS?	
a. Yes	79%
b. No	21%
12. How many EUS do you perform yearly? (n = 21)	
a. <50 per year	5%
b. 50-100 per year	5%
c. 100-200 per year	9%
d. >200 per year	82%
Diagnostic workup	
1. Do you get patients referred to perform an endoscopic papillectomy?	
a. Yes	100%
b. No	0%
2. Which diagnostic modalities are usually already performed by the referring physician?	
a. CT	50%
b. EUS	29%
c. MRCP	29%
d. Cholangiography	7%
e. None of the above	25%
f. Other	
i. Gastroduodenoscopy	29%
3. What are possible reasons to perform a CT before resection?	
a. Part of the standard diagnostic workup	43%
b. Jaundice	64%
c. Cholestatic laboratory features, without clinical signs of jaundice	43%
d. Significant weight loss	57%
e. Double duct sign on other imaging	61%
f. Age older than 50	11%
g. Large (>2 cm) adenoma	36%
h. Endoscopic features of possible malignancy	79%
i. Other	7%
4. What are possible reasons to perform an MRCP before resection?	
a. Part of the standard diagnostic workup	46%
b. Jaundice	71%
c. Cholestatic laboratory features, without clinical signs of jaundice	68%
d. Significant weight loss	36%

(*continued on the next page*)

SUPPLEMENTARY TABLE 1. Continued

Question	Agreement
e. Double duct sign on other imaging	57%
f. Age older than 50	7%
g. Large (>2 cm) adenoma	43%
h. Endoscopic features of possible malignancy	54%
i. Other	11%
5. What are possible reasons to perform an EUS before resection?	
a. Part of the standard diagnostic workup	50%
b. Jaundice	61%
c. Cholestatic laboratory features, without clinical signs of jaundice	57%
d. Significant weight loss	39%
e. Double duct sign on other imaging	61%
f. Age older than 50	11%
g. Large (>2 cm) adenoma	54%
h. Endoscopic features of possible malignancy	75%
i. Other	14%
6. What are possible reasons to perform a cholangiography before resection?	
a. Part of the standard diagnostic workup	25%
b. Jaundice	54%
c. Cholestatic laboratory features, without clinical signs of jaundice	29%
d. Significant weight loss	14%
e. Double duct sign on other imaging	50%
f. Age older than 50	4%
g. Large (>2 cm) adenoma	14%
h. Endoscopic features of possible malignancy	50%
i. Other	21%
7. Do you routinely perform biopsy sampling before resection?	
a. Yes	75%
b. No	25%
Lesion assessment and staging	
1. Do you use any kind of predefined classification system to determine if a lesion of the ampulla of Vater is most likely benign or malignant?	
a. Yes	11%
b. No	89%
2. Which of the following characteristics on endoscopic imaging do you normally take into account to determine if the lesion is benign or malignant?	
a. Presence of ulceration	100%
b. Aspect of the surface (granular vs smooth)	43%
c. Friability	61%
d. Firmness/rigidity	82%
e. Mobility	61%
f. Spontaneous bleeding	54%
g. Tumor size	57%
h. Umbilicated lesion	46%
i. Other	0%

(*continued on the next page*)

SUPPLEMENTARY TABLE 1. Continued

Question	Agreement
3. Is there a specific tumor size that would raise your suspicion of the lesion being malignant?	
a. Tumor size does not raise the suspicion of being malignant	54%
b. >1 cm	0%
c. >2 cm	18%
d. >3 cm	21%
e. >4 cm	7%
4. Do you believe any of the characteristics mentioned below is specific enough by itself to define a lesion as most likely malignant? If yes, which?	
a. Ulceration	79%
b. Smooth surface	4%
c. Excessive friability	18%
d. Firm consistency	32%
e. Immobility	36%
f. Spontaneous bleeding	14%
g. Tumor size >4 cm	14%
h. No, only a combination of the characteristics makes it possible to define a lesion as most likely malignant	21%
i. Other	4%
5. What are possible reasons to refer the patient for surgical management instead of performing endoscopic papillectomy?	
a. Jaundice (without evident signs of malignancy based on endoscopic imaging)	14%
b. Ingrowth CBD ≤1 cm	14%
c. Ingrowth PD ≤1 cm	21%
d. Ingrowth CBD >1 cm	75%
e. Ingrowth PD >1 cm	86%
f. Tumor size >4 cm	18%
g. Other	18%
6. Do you use any advanced imaging techniques to distinguish adenomatous from nonadenomatous tissue?	
a. Yes, always	29%
b. Yes, sometimes	36%
c. No, never	36%
7. Do you look for signs of central retraction, also called an umbilicated lesion?	
a. Yes, this raises the suspicion of malignancy and patient should be referred for surgical management	18%
b. Yes, but the lesion could still be removed endoscopically if there are no further signs of malignancy	75%
c. No	7%
8. Considering a fit, 70-year-old patient without any significant comorbidities, how would you treat the lesion if biopsy before resection shows only adenomatous tissue with low-grade dysplasia but endoscopic imaging shows possible signs of malignancy?	
a. Endoscopically resect the lesion based on the biopsy sample result, disregarding the endoscopic imaging. Only refer the patient for surgery if the resected specimen shows malignancy.	82%
b. Refer the patient for surgical management.	18%
9. When there is ingrowth in the CBD of more than 1 cm, would you consider endoscopic papillectomy in combination with RFA?	
a. Yes	50%
b. No, I would refer the patient for surgical treatment	50%
Technical aspects	
1. Where do you perform the resection of the lesion?	
a. At the plane of the duodenal wall	100%
b. Just above the plane of the duodenal wall	0%

(*continued on the next page*)

SUPPLEMENTARY TABLE 1. Continued

Question	Agreement
2. What type of electrosurgical current do you use most often while performing endoscopic papillectomy?	
a. Pure coagulation current	0%
b. Pure cutting current	14%
c. Fractionated current (short regular pulses of cutting current integrated in background of coagulation current)	79%
d. Other	7%
3. Do you standard use submucosal injection before endoscopic papillectomy?	
a. Yes	4%
b. No, only in case of lateral spreading	79%
c. No	18%
4. Do you routinely perform pancreatic sphincterotomy?	
a. Yes, always	14%
b. Yes, sometimes	36%
c. No, never	50%
5. When you perform pancreatic sphincterotomy do you in general perform it before or after resection? (n = 14)	
a. Before resection	0%
b. After resection	100%
6. Do you routinely place a stent in the PD to prevent postintervention pancreatitis?	
a. Yes	89%
b. Only if PD drainage is deemed suboptimal or if the PD is difficult to cannulate after the procedure	11%
c. No	0%
7. Do you cannulate the PD before or after resection?	
a. Cannulate before resection and perform the resection with guidewire inside the PD.	7%
b. Inject the PD before resection and cannulate it after resection.	43%
c. Cannulate after resection.	50%
8. What kind of PD stent do you use in general?	
a. Stent with an internal flap	46%
b. Stent without an internal flap	54%
9. Do you routinely perform biliary sphincterotomy?	
a. Yes, always	32%
b. Only in case of concomitant bile duct stones	57%
c. No, never	11%
10. When you perform biliary sphincterotomy do you in general perform it before or after resection? (n = 25)	
a. Before resection	4%
b. After resection	96%
11. Do you place a stent in the common bile duct after resection as standard practice?	
a. Yes	18%
b. No, only on indication	71%
c. No, never	11%
12. What are the indications to place a stent in the CBD after resection? (n = 20)	
a. Bleeding during the procedure	80%
b. Residual adenomatous tissue	70%
c. Concerns for microperforations after resection	80%

(*continued on the next page*)

SUPPLEMENTARY TABLE 1. Continued

Question	Agreement
d. Advanced resection	25%
e. Multiple submucosal injections	5%
f. Extensive piecemeal resection	20%
g. Other	30%
13. What type of biliary stent do you prefer if the bile duct is 8 mm in diameter? (n = 25)	
a. Plastic	72%
b. FCSEMS	28%
14. What adjunct modality do you use most commonly to remove small (<1 cm) residual tissue during index procedure after papillectomy?	
a. Cold forceps biopsy	32%
b. Argon plasma coagulation	43%
c. Monopolar/multipolar coagulation probe	4%
d. Snare tip soft coagulation	36%
e. Other	18%
15. Do you use snare tip soft coagulation of the margins after resection?	
a. Yes	0%
b. Yes, but only in case of lateral spread	25%
c. No	75%
16. Would you perform RFA when EUS shows ingrowth in the CBD of <1 cm?	
a. Yes	50%
b. No	50%
Complications and management	
1. Do you standard give rectal indomethacin/diclofenac suppository before resection to prevent postintervention pancreatitis?	
a. Yes	75%
b. No	25%
2. Do you use vigorous hydration to prevent postintervention pancreatitis?	
a. Yes	54%
b. No	46%
3. Do you routinely start patients on PPI after performing an endoscopic papillectomy?	
a. Yes	50%
b. No, only on indication	32%
c. No, never	18%
4. What are the indications to start patients on PPI after performing an endoscopic papillectomy?	
a. Excessive friability of tissue during the procedure	11%
b. Use of antithrombotic medication	33%
c. Advanced resection	67%
d. Multiple submucosal injections	0%
e. Extensive piecemeal resection	67%
f. Other	11%
5. For how long will you normally treat patients with PPI after performing an endoscopic papillectomy?	
a. 24 hours	9%
b. Between 24 hours and 2 weeks	39%
c. Between 2 weeks and 1 month	52%
d. More than 1 month	0%

(*continued on the next page*)

SUPPLEMENTARY TABLE 1. Continued

Question	Agreement
6. If bleeding occurs after endoscopic papillectomy and the patient is hemodynamic stable after resuscitation with <2 g/dL drop in hemoglobin, how would you manage it initially?	
a. Continue PPI, but no endoscopic intervention because patient is stable.	25%
b. Endoscopic reintervention to treat bleeding.	64%
c. Other	11%
Follow-up	
1. After which period would you perform the first endoscopic follow-up?	
a. 3 months	54%
b. 3-6 months	4%
c. 6 months	18%
d. 12 months	4%
e. Other	21%
2. Which follow-up interval do you use if no residual adenomatous tissue is seen at first follow-up endoscopy?	
a. 6 months	46%
b. 12 months	39%
c. >12 months	4%
d. Other	11%
3. Until when would you perform endoscopic follow-up?	
a. Up to 2 years after endoscopic papillectomy	11%
b. Up to 3 years after endoscopic papillectomy	14%
c. Up to 5 years after endoscopic papillectomy	32%
d. Up to 10 years after endoscopic papillectomy	7%
e. Lifelong, as long as patient is fit	32%
f. Other	4%
4. Do you take biopsy samples during follow-up as standard practice?	
a. Yes	36%
b. No, only when macroscopic abnormalities are present	64%
c. No, never	0%

CBD, Common bile duct; *CT*, computed tomography; *EMR*, endoscopic mucosal resection; *ERCP*, endoscopic retrograde cholangiopancreatography; *EUS*, endoscopic ultrasound; *FCSEMS,* fully covered self-expanding metal stent; *MRCP*, magnetic resonance cholangiopancreatography; *MRI*, magnetic resonance imaging; *PD,* pancreatic duct; *PPI,* proton pump inhibitor; *RFA,* radiofrequency ablation.

SUPPLEMENTARY TABLE 2. Survey round 2

Question	Agreement
Diagnostic workup	
Please fill out the order in which you believe the different diagnostic modalities should be performed for different indications (1 = first choice, 2 = only if test(s) of first choice are inconclusive, 3 = only if test(s) of second choice are inconclusive, 4 = only if test(s) of third choice are inconclusive, 5 = only if all earlier performed test(s) are inconclusive, 6 = never)	
1. In every patient	
a. CT	3 (IQR 4)
b. MRCP	2 (IQR 2)
c. EUS	2 (IQR 1)
d. Endoscopic cholangiography	4 (IQR 2)
e. Biopsy sampling	1 (IQR 0)
2. In case of jaundice	
a. CT	2 (IQR 2)
b. MRCP	2 (IQR 1)
c. EUS	2 (IQR 1)
d. Endoscopic cholangiography	3 (IQR 3)
e. Biopsy sampling	1 (IQR 0)
3. In case of cholestatic laboratory features without clinical signs of jaundice	
a. CT	2 (IQR 1)
b. MRCP	1 (IQR 1)
c. EUS	2 (IQR 1)
d. Endoscopic cholangiography	3 (IQR 2)
e. Biopsy sampling	1 (IQR 1)
4. In case of significant weight loss	
a. CT	1 (IQR 1)
b. MRCP	2 (IQR 2)
c. EUS	2 (IQR 2)
d. Endoscopic cholangiography	4 (IQR 2)
e. Biopsy sampling	1 (IQR 1)
5. In case of double duct sign on other imaging	
a. CT	2 (IQR 1)
b. MRCP	2 (IQR 1)
c. EUS	1 (IQR 1)
d. Endoscopic cholangiography	3 (IQR 2)
e. Biopsy sampling	1 (IQR 0)
6. In case patient is older than 50 years	
a. CT	2 (IQR 2)
b. MRCP	2 (IQR 2)
c. EUS	2 (IQR 1)
d. Endoscopic cholangiography	4 (IQR 2)
e. Biopsy sampling	1 (IQR 0)
7. In case of large (>2 cm) adenoma	
a. CT	2 (IQR 2)
b. MRCP	2 (IQR 1)
c. EUS	1 (IQR 1)
d. Endoscopic cholangiography	4 (IQR 2)
e. Biopsy sampling	1 (IQR 0)

(*continued on the next page*)

SUPPLEMENTARY TABLE 2. Continued

Question	Agreement
8. In case of endoscopic signs of malignancy	
a. CT	1 (IQR 1)
b. MRCP	2 (IQR 1)
c. EUS	2 (IQR 1)
d. Endoscopic cholangiography	3 (IQR 2)
e. Biopsy sampling	1 (IQR 0)
9. Do you believe pancreas divisum should be ruled out in every patient before resection?	
a. Yes	65%
b. No	35%
10. Could you please fill out in which order the following diagnostic modalities should be performed to rule out pancreas divisum before resection? (1 = first choice, 2 = only if first choice is inconclusive, 3 = only if earlier performed test(s) are found inconclusive, 4 = never)	
a. MRCP	2 (IQR 0)
b. EUS	2 (IQR 0)
c. Endoscopic cholangiography	3 (IQR 1)
11. Do you believe local extension should be ruled out in every patient before resection?	
a. Yes	53%
b. Yes, except for small (<2 cm) adenoma or in case of familial adenomatous polyposis	29%
c. No	18%
12. Could you please fill out in which order the following diagnostic modalities should be performed to rule out local extension before resection? (1 = first choice, 2 = only if first choice is inconclusive, 3 = only if earlier performed test(s) are found inconclusive, 4 = never)	
a. MRCP	2 (IQR 1)
b. EUS	1 (IQR 0)
c. Endoscopic cholangiography	2 (IQR 1)
Lesion assessment and staging	
1. When a lesion shows ulceration, this lesion should be defined as most likely malignant.	94%
2. The following characteristics are *not* a reason to define the lesion as most likely malignant	
a. Smooth surface	71%
b. Spontaneous bleeding	53%
c. Lesion size >4 cm	35%
3. You should refer a patient for surgical management in case of	
a. Ingrowth in the PD >1 cm	76%
b. Ingrowth in the CBD >1 cm	47%
4. The following situations are *not* a reason to refer the patient for surgical management	
a. Jaundice	41%
b. Ingrowth in the PD ≤1 cm	41%
c. Ingrowth in the CBD ≤1 cm	65%
d. An umbilicated lesion	53%
5. When a lesion shows ulceration, do you believe this patient should be referred for surgical management? (n = 16)	
a. Yes, regardless of possible biopsy sampling results	38%
b. Only when biopsy specimen shows HGD	63%

(*continued on the next page*)

SUPPLEMENTARY TABLE 2. Continued

Question	Agreement
6. If a patient is not a surgical candidate because of age and/or comorbidity, in which of the following situations would you still resect the lesion endoscopically?	
a. There is ingrowth of >1 cm in the CBD	59%
b. There is ingrowth of >1 cm in the PD	35%
c. The lesion shows ulceration	35%
d. Biopsy sample shows adenocarcinoma	47%
e. In none of the above described situations	29%
f. Other	0%
7. When there is ingrowth in the CBD of >1 cm, would you consider endoscopic papillectomy with RFA?	
a. In a fit 60-year-old patient	47%
b. In a patient who is not a surgical candidate because of age and/or comorbidity	76%
8. Do you believe any advanced imaging techniques (such as narrow-band imaging or chromoendoscopy) would be helpful to distinguish between benign and malignant lesions?	
a. Yes	29%
b. No	71%
9. If the biopsy sample shows LGD, which of the following characteristics would make you to refer the patient for surgical management? (0 = would not take this into account, 5 = would refer patient for surgical treatment based on the sole characteristic)	
a. Ulceration	4 (IQR 1)
b. Smooth or irregular surface	2 (IQR 2)
c. Excessive friability	1 (IQR 3)
d. Firm consistency	3 (IQR 2)
e. Immobility	4 (IQR 1)
f. Spontaneous bleeding	2 (IQR 3)
g. Tumor size >4 cm	2 (IQR 2)
10. If the biopsy sample shows HGD, which of the following characteristics would make you to refer the patient for surgical management?	
a. Ulceration	4 (IQR 1)
b. Smooth or irregular surface	3 (IQR 3)
c. Excessive friability	4 (IQR 3)
d. Firm consistency	4 (IQR 1)
e. Immobility	4 (IQR 0)
f. Spontaneous bleeding	4 (IQR 2)
g. Tumor size >4 cm	3 (IQR 2)
Technical aspects	
1. Resection of the lesion should be performed at the plane of the duodenal wall.	94%
2. Endoscopic papillectomy should be performed with fractionated current.	94%
3. Submucosal injection should only be performed in case of a laterally spreading lesion.	88%
4. If pancreatic sphincterotomy is performed, then it should be performed after resection.	88%
5. PD stent should be placed routinely to prevent postintervention pancreatitis.	100%
6. PD should be cannulated after resection.	100%
7. If biliary sphincterotomy is performed, it should be performed after resection.	100%
8. A CBD stent should only be placed on indication.	82%
9. A CBD stent should be placed in the following cases:	
a. In case of bleeding during the procedure.	76%
b. If there are any concerns for microperforations.	88%
c. In case of residual adenomatous tissue.	59%

(*continued on the next page*)

SUPPLEMENTARY TABLE 2. Continued

Question	Agreement
10. STSC of the margins after endoscopic papillectomy should *not* be performed.	65%
11. When do you believe a pancreatic sphincterotomy should be performed?	
a. Never	29%
b. In case of intrapancreatic duct extent	59%
c. In case of pancreas divisum instead of PD stent	6%
d. Other	12%
12. Do you believe it is better to inject the PD before resection to make it easier to find the PD after resection?	
a. Yes	41%
b. No	59%
13. Which kind of PD stent would you choose in the following situations?	
a. Always with an internal flap	47%
b. Always without an internal flap	35%
c. Without an internal flap, but in case of large (>2 cm) lesions	0%
d. Without an internal flap, but in case of perforation	18%
e. Without an internal flap, but after coagulation or RFA	18%
f. Other	0%
14. When would you perform biliary sphincterotomy?	
a. Always	18%
b. In case of bile duct stones	76%
c. In case of drainage is deemed suboptimal	53%
d. Other	6%
15. Which stent do you prefer in a bile duct with diameter ≤8 mm in the following cases	
a. Bleeding during the procedure	
i. Plastic	41%
ii. FCSEMS	59%
iii. No stent	0%
b. Concerns for microperforations after resection	
i. Plastic	35%
ii. FCSEMS	65%
iii. No stent	0%
c. Residual tissue	
i. Plastic	29%
ii. FCSEMS	71%
iii. No stent	0%
16. Which stent do you prefer in a bile duct with diameter >8 mm in the following cases	
a. Bleeding during the procedure	
i. Plastic	24%
ii. FCSEMS	65%
iii. No stent	12%
b. Concerns for microperforations after resection	
i. Plastic	18%
ii. FCSEMS	71%
iii. No stent	12%

(*continued on the next page*)

SUPPLEMENTARY TABLE 2. Continued

Question	Agreement
c. Residual tissue	
i. Plastic	41%
ii. FCSEMS	35%
iii. No stent	24%
17. What adjunct modalities would you consider to remove small (<1 cm) residual tissue during index procedure after papillectomy?	
a. Cold forceps biopsy sampling	65%
b. APC	53%
c. Monopolar/multipolar coagulation probe	12%
d. STSC	18%
e. Hot snare	41%
f. Cold snare	53%
g. Other	6%
18. How would you resect the lesion when EUS shows ingrowth in the CBD of <1 cm?	
a. Snare resection in combination with RFA	24%
b. Snare resection in combination with APC	35%
c. Resect intraductal residue in second procedure	47%
d. Resect intraductal residue in second procedure after placement of FCSEMS during first procedure	18%
e. Other	6%
19. Do you standardly use clips to close the defect after resection?	
a. Yes	41%
b. No	59%
20. Do you standardly give buscopan or glucagon before resection to reduce the risk of loss of specimen further in the GI tract?	
a. Yes	59%
b. No	41%
21. Would you use a different current to resect the lesion based on the size of the lesion?	
a. Large (>2 cm) lesion	
i. Pure coagulation current	0%
ii. Pure cutting current	6%
iii. Fractionated current	94%
b. Small (<2 cm) lesion	
i. Pure coagulation current	6%
ii. Pure cutting current	24%
iii. Fractionated current	71%
Complications and management	
1. Rectal indomethacin or diclofenac should be given before resection.	82%
2. If bleeding occurs during intervention, which modalities to stop the bleeding would you consider?	
a. FCSEMS	71%
b. Coagulation grasper	65%
c. Goldprobe	12%
d. Hemospray	41%
e. STSC	24%
f. APC	12%
g. Other	35%

(*continued on the next page*)

SUPPLEMENTARY TABLE 2. Continued

Question	Agreement
3. When would you start patients on PPI after performing an endoscopic papillectomy?	
a. Always	65%
b. Extensive friability of the tissue during the procedure	6%
c. Use of antithrombotic medication	0%
d. Advanced resection of large lesion	24%
e. Multiple submucosal injections	6%
f. Extensive piecemeal resection	18%
g. Never	12%
4. How long do you believe patients should be treated with PPI after resection?	
a. 24 hours	6%
b. 48 hours	0%
c. 1 week	24%
d. 2 weeks	18%
e. 3 weeks	0%
f. 1 month	47%
5. If a bleeding occurs after endoscopic papillectomy and the patient is hemodynamic stable after resuscitation with <2-g/dL drop in hemoglobin, how would you manage it initially?	
a. Expectative (continue or start PPI), no endoscopic intervention because patient is stable	41%
b. Endoscopic reintervention to treat bleeding within working hours	47%
c. Endoscopic reintervention to treat bleeding as an emergency procedure	12%
Follow-up	
1. Do you standardly admit patients for observation after endoscopic papillectomy? If yes, how long, if no complications occur?	
a. 6 hours	0%
b. 24 hours	29%
c. 48 hours	35%
d. Other	
i. No	18%
ii. >48 hours	18%
2. After which period should first follow-up be performed after presumed complete removal (excluding the removal of possible placed stents within 1-3 months)?	
a. In case initial pathology shows LGD	
i. 3 months	35%
ii. 3-6 months	29%
iii. 6 months	24%
iv. 6-12 months	12%
v. >12 months	0%
b. In case initial pathology shows HGD	
i. 3 months	76%
ii. 3-6 months	24%
iii. 6 months	0%
iv. 6-12 months	0%
v. >12 months	0%

(*continued on the next page*)

SUPPLEMENTARY TABLE 2. Continued

Question	Agreement
3. What should be the follow-up interval if no residual tissue is seen at first follow-up?	
a. In case initial pathology shows LGD	
i. 6 months	35%
ii. 12 months	53%
iii. >12 months	12%
b. In case initial pathology shows HGD	
i. 6 months	65%
ii. 12 months	35%
iii. >12 months	0%
4. Until when should endoscopic follow-up be performed?	
a. In case initial pathology shows LGD	
i. Up to 2 years	12%
ii. Up to 3 years	12%
iii. Up to 5 years	41%
iv. Up to 10 years	12%
v. Lifelong, as long as patient is fit	24%
b. In case initial pathology shows HGD	
i. Up to 2 years	12%
ii. Up to 3 years	0%
iii. Up to 5 years	47%
iv. Up to 10 years	6%
v. Lifelong, as long as patient is fit	35%
5. Should biopsy samples be taken routinely at first follow-up?	
a. In case initial pathology shows LGD	
i. Yes	29%
ii. No, only when macroscopic abnormalities are present	71%
b. In case initial pathology shows HGD	
i. Yes	35%
ii. No, only when macroscopic abnormalities are present	65%
6. Should biopsy samples be taken routinely at further follow-up?	
a. In case initial pathology shows LGD	
i. Yes	18%
ii. No, only when macroscopic abnormalities are present	82%
b. In case initial pathology shows HGD	
i. Yes	35%
ii. No, only when macroscopic abnormalities are present	65%

CBD, Common bile duct; *CT*, computed tomography; *EUS*, endoscopic ultrasound; *FCSEMS,* fully covered self-expanding metal stent; *IQR*, interquartile range; *HGD*, high-grade dysplasia; *LGD*, low-grade dysplasia; *MRCP*, magnetic resonance cholangiopancreatography; *MRI*, magnetic resonance imaging; *STSC*, snare tip soft coagulation; *APC*, argon plasma coagulation; *PD,* pancreatic duct; *PPI,* proton pump inhibitor; *RFA,* radiofrequency ablation.

SUPPLEMENTARY TABLE 3. Survey round 3

Question	Agreement
Diagnostic workup statements	
1. Gastroduodenoscopy (with side-viewing instrument) should always be performed before resection.	100%
2. Biopsy sampling should always be performed before resection.	94%
3. Either MRI/MRCP or EUS should be performed in every patient before resection.	63%
4. Either MRI/MRCP or EUS should be performed in case of cholestatic laboratory features with or without jaundice.	81%
5. Either CT, MRI/MRCP or EUS should be performed in case of cholestatic laboratory features with or without jaundice.	75%
6. CT should be performed in case of jaundice.	75%
7. Either MRI/MRCP or EUS should be performed in case of a lesion larger than 2 cm.	75%
8. Either MRI/MRCP or CT should be performed in case of significant weight loss.	81%
9. Either EUS, MRI/MRCP, or CT should be performed in case of significant weight loss.	63%
10. Either MRI/MRCP or CT should be performed in case of endoscopic signs of malignancy.	81%
11. Either CT or EUS should be performed in case of endoscopic signs of malignancy.	69%
12. Either MRI/MRCP, EUS, or CT should be performed in case of endoscopic signs of malignancy.	81%
13. An endoscopic cholangiogram either before or during endoscopic papillectomy should only be performed if other performed tests are found inconclusive and there is still doubt about the presence of intraductal extension.	44%
Lesion assessment and staging	
1. Patient should be referred for surgical management in case of ingrowth in the CBD of more than 1 cm, considering patient is suitable for surgery.	81%
2. If biopsy sample shows LGD and ulceration is present, the lesion could still be resected endoscopically; there is no need to refer the patient for surgical management based on this sole characteristic, considering the lesion seems favorable for endoscopic resection.	88%
3. If biopsy sample shows HGD and ulceration is present, the lesion could still be resected endoscopically; there is no need to refer the patient for surgical management based on this sole characteristic, considering the lesion seems favorable for endoscopic resection.	63%
4. If there is ingrowth in the CBD of more than 1 cm, endoscopic resection can still be considered if the patient is not a surgical candidate because of age and/or comorbidity, considering the lesion seems favorable for endoscopic resection.	81%
5. If biopsy sample shows adenocarcinoma in situ or well-differentiated adenocarcinoma, endoscopic resection can still be considered if the patient is not a surgical candidate because of age and/or comorbidity, considering the lesion seems favorable for endoscopic resection.	75%
6. If there is ingrowth in the CBD of more than 1 cm, endoscopic papillectomy with RFA should *not* be performed in a fit 60-year-old patient.	44%
7. If there is ingrowth in the CBD of more than 1 cm, endoscopic papillectomy with RFA can be considered in a patient that is not a surgical candidate because of age and/or comorbidity, considering the lesion seems favorable for endoscopic resection.	75%
Technical aspects	
1. Snare tip soft coagulation of the margins should *not* be performed after endoscopic papillectomy.	56%
2. Snare tip soft coagulation can be performed for the margins of the laterally spreading component, but not the papillary margins.	50%
3. Pancreatic sphincterotomy after resection should only be performed in case of extension in the pancreatic duct.	38%
4. Pancreatic sphincterotomy after resection should be performed in case of extension in the pancreatic duct or if drainage is deemed suboptimal.	44%
5. It can be helpful to inject the PD before resection to make it easier to find the PD after resection in case of extension in the pancreatic duct.	44%
6. Biliary sphincterotomy should be performed only in case of concomitant bile duct stones.	44%
7. Biliary sphincterotomy should be performed in case of concomitant bile duct stones and in case drainage is deemed suboptimal.	81%
8. In case there is bleeding during the procedure, an FCSEMS instead of a plastic stent should be placed in the CBD.	63%
9. In case there are concerns for microperforations in the papillary region, an FCSEMS should be placed in the CBD.	88%

(*continued on the next page*)

SUPPLEMENTARY TABLE 3. Continued

Question	Agreement
10. In case there are concerns for residual adenomatous tissue in the distal part of the CBD, an FCSEMS should be placed in the CBD.	31%
11. In case there are concerns for residual adenomatous tissue in the distal part of the CBD, either a plastic stent or FCSEMS should be placed in the CBD.	44%
12. Please rank the following items in which order you would consider them if there is ingrowth in the CBD of <1 cm, which seems accessible from the duodenum. (1 = first choice, 5 = never)	
a. Snare resection in combination with RFA	2 (IQR 1.5)
b. Snare resection in combination with APC	3 (IQR 3.25)
c. Resect intraductal residue in second procedure after placement of FCSEMS during first procedure	3 (IQR 1.25)
d. Resect with snare inside duct	2.5 (IQR 2.25)
13. Standard clip closure of the mucosal defect after resection should *not* be performed.	38%
14. Glucagon or buscopan should be provided routinely before resection to reduce the risk of losing the specimen into the GI tract.	56%
Adverse events and management	
1. Vigorous hydration should be considered in patients without any cardiologic comorbidity to further decrease the risk of postintervention pancreatitis.	63%
Please rank the following methods in which order you would consider them to use in case of intraprocedural bleeding in the following hypothetical situations (1 = first choice, 6 = never)	
2. In case of bleeding from the papillectomy site (not from lateral spreading component) considering the PD is already protected by a stent.	
a. FCSEMS	3.5 (IQR 1.5)
b. Hemospray	5 (IQR 2)
c. Epinephrine	2 (IQR 1.25)
d. Clips	3 (IQR 1)
e. Coagulation probe (or other instrument used to coagulate)	2 (IQR 1)
3. In case of bleeding from the papillectomy site (not from lateral spreading component) however the PD is not yet protected by a stent.	
a. FCSEMS	4 (IQR 2.25)
b. Hemospray	4 (IQR 3)
c. Epinephrine	1 (IQR 1)
d. Clips	3 (IQR 3.25)
e. Coagulation probe (or other instrument used to coagulate)	2 (IQR 2)
4. In case of bleeding from the lateral spreading component.	
a. FCSEMS	5.5 (IQR 1)
b. Hemospray	4 (IQR 2)
c. Epinephrine	2 (IQR 2)
d. Clips	2.5 (IQR 2)
e. Coagulation probe (or other instrument used to coagulate)	2 (IQR 1)
5. Every patient should be treated with PPI after performing an endoscopic papillectomy.	69%
6. Patients treated with PPI after resection should be treated for at least 2 weeks.	69%
7. Patients treated with PPI after resection should be treated for at least 1 month.	56%
8. If a bleeding occurs after endoscopic papillectomy and the patient is hemodynamic stable after resuscitation with <2-g/dL drop in hemoglobin, reintervention should be performed within 12 hours.	38%
9. If a bleeding occurs after endoscopic papillectomy and the patient is hemodynamic stable after resuscitation with <2-g/dL drop in hemoglobin, conservative treatment (continue or start PPI) is initially indicated.	63%

(*continued on the next page*)

SUPPLEMENTARY TABLE 3. Continued

Question	Agreement
Follow-up	
1. Every patient should be admitted for at least 24 hours for observation after endoscopic papillectomy.	69%
2. Every patient should be admitted for at least 48 hours for observation after endoscopic papillectomy.	44%
3. In case initial pathology shows LGD	
a. First follow-up (after removal of possible placed stents) should be performed within 6 months.	81%
b. First follow-up (after removal of possible placed stents) should be performed within 3 months.	56%
c. At first follow-up biopsy samples should only be taken when macroscopic abnormalities are present.	94%
d. Follow-up interval should be 12 months or less.	88%
e. Follow-up interval should be 6 months or less.	38%
f. At further follow-up biopsy samples should only be taken when macroscopic abnormalities are present.	94%
g. Follow-up should be performed for at least 5 years.	81%
h. Follow-up should be performed lifelong, as long as patient is fit.	31%
4. In case initial pathology shows HGD	
a. First follow-up (after removal of possible placed stents) should be performed within 6 months.	31%
b. First follow-up (after removal of possible placed stents) should be performed within 3 months.	94%
c. At first follow-up biopsy samples should only be taken when macroscopic abnormalities are present.	81%
d. Follow-up interval should be 12 months or less.	25%
e. Follow-up interval should be 6 months or less.	94%
f. At further follow-up biopsy samples should only be taken when macroscopic abnormalities are present.	81%
g. Follow-up should be performed for at least 5 years.	75%
h. Follow-up should be performed lifelong, as long as patient is fit.	38%

CBD, Common bile duct; *CT*, computed tomography; *EUS*, endoscopic ultrasound; *MRCP*, magnetic resonance cholangiopancreatography; *MRI*, magnetic resonance imaging; *FCSEMS,* fully covered self-expanding metal stent; *HGD*, high-grade dysplasia; *LGD*, low-grade dysplasia; *STSC*, snare tip soft coagulation; *APC*, argon plasma coagulation; *PD,* pancreatic duct; *PPI,* proton pump inhibitor; *RFA,* radiofrequency ablation.
